# Patterns of Service Provision in Older People’s Mental Health Care in Australia

**DOI:** 10.3390/ijerph17228516

**Published:** 2020-11-17

**Authors:** Hossein Tabatabaei-Jafari, Jose A. Salinas-Perez, Mary Anne Furst, Nasser Bagheri, John Mendoza, David Burke, Peter McGeorge, Luis Salvador-Carulla

**Affiliations:** 1 Centre for Mental Health Research, Australian National University, Canberra, ACT 2601, Australia; hossein.tabatabaei@anu.edu.au (H.T.-J.); mary.furst@anu.edu.au (M.A.F.); nasser.bagheri@anu.edu.au (N.B.); luis.salvador-carulla@anu.edu.au (L.S.-C.); 2 Department of Quantitative Methods, Universidad Loyola Andalucía, 41704 Dos Hermanas, Sevilla, Spain; 3 Mental Health & Prison Health, Central Adelaide Local Health Network, Adelaide, SA 5000, Australia; John.Mendoza@sa.gov.au; 4 Brain and Mind Centre, University of Sydney, Sydney, NSW 2050, Australia; 5 Discipline of Psychiatry, University of Notre Dame, Sydney, NSW 2010, Australia; david.burke@svha.org.au (D.B.); peter.mcgeorge@gmail.com (P.M.); 6 School of Psychiatry, University of New South Wales, Sydney, NSW 2052, Australia; 7 Menzies Centre for Health Policy, University of Sydney, Sydney, NSW 2006, Australia

**Keywords:** psychogeriatric, older people’s mental health service, integrated atlas, DESDE-LTC, healthcare ecosystem, health planning

## Abstract

Australia has a population of around 4 million people aged 65 years and over, many of whom are at risk of developing cognitive decline, mental illness, and/or psychological problems associated with physical illnesses. The aim of this study was to describe the pattern of specialised mental healthcare provision (availability, placement capacity, balance of care and diversity) for this age group in urban and rural health districts in Australia. The Description and Evaluation of Services and DirectoriEs for Long Term Care (DESDE-LTC) tool was used in nine urban and two rural health districts of the thirty-one Primary Health Networks across Australia. For the most part service provision was limited to hospital and outpatient care across all study areas. The latter was mainly restricted to health-related outpatient care, and there was a relative lack of social outpatient care. While both acute and non-acute hospital care were available in urban areas, in rural areas hospital care was limited to acute care. Limited access to comprehensive mental health care, and the uniformity in provision across areas in spite of differences in demographic, socioeconomic and health characteristics raises issues of equity in regard to psychogeriatric care in this country. Comparing patterns of mental health service provision across the age span using the same classification method allows for a better understanding of care provision and gap analysis for evidence-informed policy.

## 1. Introduction

A growing proportion of the population is surviving longer and thus becoming vulnerable to decline in cognition and to mental disorders associated with medical comorbidities [1,2]. Indeed, the prevalence of cognitive impairment and dementia affects more than 40% of those aged 80 years and over [3]; and mental disorders are the third leading cause (after musculoskeletal disorders and other non-communicable disorders) of the burden of disease for people aged 65 to 74 in Australia [4]. The overall prevalence of depression is estimated to be more than 25% in this population group [5]; with suicide rates steadily increasing with age [6].

During the last decade, a number of international organisations have made a call for an integrated model of care for older people covering specific interventions for different disorders and a complex array of service provision settings including homecare, community, hospital, and other residential settings [7,8]. There is a demand to improve the productivity and quality of care services and to fully integrating the health care and social elements of long-term care provision for the elderly to boost efficient and equitable care provision [8]. An integrated perspective is essential in care for older people by the close relationship between the capacity of residential aged care services and the rate of hospital discharge [9], COVID-19 has shown the vulnerability of this community residential care subsystem, its connectedness to hospital care [10], and the importance of better international comparisons encompassing all relevant care sectors [11].

Improving healthcare and social services for older people is a priority in Australia [12], although older people’s mental health has not been included as a specific strategic goal. Psychiatric services for dementia are unevenly distributed across Australia, with the information aggregated at a state level, focussed on public health services [13], and lacking a whole system perspective [14]. 

The need of more health service research in this area has been pointed out as a main priority including a wider use of statistical instruments to monitor resource information, service provision and quality of services to support informed policy decision making [7]. However, there are major challenges for producing standard and valid comparison of the patterns of care across different jurisdictions [15], particularly in the care of older people [16]. National and international comparisons are hindered by ambiguity and inconsistency in service definition and description; differences in organisational structure and complexity of service networks; and differences in the definition of the target population [17]. Hence using a common assessment and coding system allows harmonisation of service data, and can inform equitable allocation of care resources, programmes, and treatments across different health districts, as well as facilitating linkages of health networks [18,19].

The Glocal Integrated Mental Health Atlas project aims to overcome these issues through the standard description of mental health services from mental healthcare ecosystems around the world. The results intend to support decision-making in mental health policy and planning through the study of the standardised service provision, the detection of gaps and redundancies, and the development of national and international comparisons. In the framework of this project, there are a number of studies analysing and mapping service provision [20,21,22,23,24,25,26,27]. However, the analysis of the pattern of care of specific services for older people had not been addressed yet.

The aim of this study was to describe the pattern of mental healthcare provision (availability, placement capacity, balance of care and diversity) for people aged 65 years and over in urban and rural health districts in Australia. 

## 2. Materials and Methods 

### 2.1. Study Desing and Procedure 

**Figure 1:**
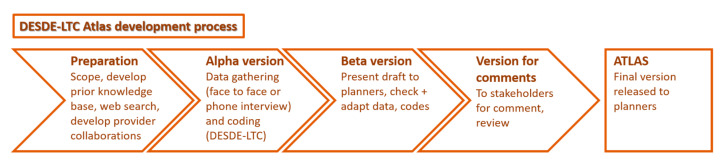
DESDE-LTC Atlas development process.

This is an ecological study for the descriptive assessment of the local patterns of care provision of specific services for the older with mental health disorders in Australia. It follows a healthcare ecosystem approach [14]. The typical procedure of the Integrated Mental Health Atlases is displayed in Figure 1.

### 2.2. Study Areas

From the 31 Primary Health Networks (PHNs) districts established by the Australian government in 2015, 11 districts (nine urban and two rural) were used in this study to map the pattern of mental health service delivery specifically for people aged 65 years and over. The study areas were described with a set of key demographic and socioeconomic indicators collected from the Social Health Atlases of Australia [28]; and the Australian Bureau of Statistics [29,30].

The standard description of these areas is part of a larger project that aims to compare patterns of mental health care in Australia and in Europe. The Australian Capital Territory (ACT), Central and Eastern Sydney, Northern Sydney, South Western Sydney, Western Sydney, Brisbane North, Eastern Melbourne, Perth North, and Perth South PHNs are urban regions, and Country Western Australia (CWA) and Western New South Wales (WNSW) are the rural and remote regions included in this study (Australian Standard Geographical Classification- Remoteness Areas -ASGC-RA) [31]. Integrated Atlases of Mental Health including all services available for the target population have been released for these PHNs (34.5% of all the PHNs in Australia) and are available at the Australian National University Integrated Health Care Atlases repository site [32].

### 2.3. Services

The Description and Evaluation of Services and DirectoriEs for Long Term Care (DESDE-LTC) [33] tool was used for the standardized description of mental health services specific to older people within the boundaries of the selected PHNs [34]. DESDE-LTC is a classification system that incorporates a standard description of all services available in a defined area using common units of analysis in service assessment, allowing comparisons across different health districts [33,34,35]. 

The DESDE-LTC has previously been used for describing care provision in over 34 countries [15]. It uses an international terminology and coding system to overcome the problem of local and national variation in the names of services. The terminology has been approved by a multidisciplinary panel of service researchers and planners, and its glossary of terms (see Appendix A) is publicly available [36]. DESDE-LTC uses a multiaxial system for the description of a service according to care teams defined as Basic Stable Inputs of Care (BSIC), which are the minimal unit of care with organisational and temporal stability (i.e., funded for more than 3 years), arranged for delivering health-related care to a defined population [33,37]. The coding of the BSIC uses a multiaxial system for the description in its catchment area of influence, the target population, and its principal activity that is identified as the Main Types of Care (MTC). There are 108 codes of MTCs, classified into six main branches:

(1) Residential care (ranging from acute wards to nursing homes and supported housing): includes services that provide beds overnight for acute or non-acute care. Acute care is care for crises due to deterioration in physical, mental, behavioural or social functioning related to the user’s health condition. Any other care is considered non-acute care. Hospitals, crisis houses, crisis hostels and emergency beds in community-based primary care setting (including those provided by non-health organisations) or mental health centres are examples of residential care.
(2) Day care (ranging from day hospitals to social clubs): provides combinations of different interventions (e.g., structured activity, or social contact and/or support) to a group of users for a period longer than a visit during the course of a day. Acute day care is care for crises due to deterioration in physical, mental, behavioural or social functioning related to their health condition. Any other care is described as non-acute care.
(3) Outpatient care (ranging from outpatient care in hospitals to community mental health centres and home-visit): provides acute and non-acute care at a point of time (care visit). Point of time interventions include face-to-face and on-line treatment for health issues and/or support for social difficulties. Services can be provided at the centre where the care team is based (non-mobile) or by outreach, including another centre or the users’ home (mobile).
(4) Accessibility of care (ranging from transportation to managed care): Facilitates access to care by identifying providers and facilitating re-engagement, facilitating communication (e.g., sign language or translation), facilitating physical mobility of users (e.g., transportation), and navigating, access, management and cohesion of treatment, care and support.
(5) Information for care (ranging from health information services to diagnostic and evaluation services): provides information and/or an assessment to users. Information provision can be interactive (such as face to face) or non-interactive such as pamphlets and webs. Assessment is not limited to health-related assessment and items such as work, education, and social and cultural support are also included. The assessment team does not provide direct care provision.
(6) Self-help and voluntary care: includes any types of care provision by unpaid peers or graduate professionals. Self-help and voluntarily services, informal care associations and teams are also included in this category.

Typically, a BSIC could be described by a single MTC, but in some cases it is necessary to include a principal main type (e.g., acute ward code R2) and an additional one (e.g., an emergency room code O3). The Mental Health Atlas project repository [32] and the systematic review of the 71 international studies using our method [15], provide information on the patterns of coding services in local areas. Over 70% of the units of production of care in different jurisdictions were described with a single MTC, and very rarely has a unit needed four codes or more to be fully described [37]. 

It is important to note that the DESDE classification system uses this coding system and its related glossary of terms to allow international comparison of service provision overcoming the existing variability in terminology [36]. A detailed description of the taxonomy and the six main types of care is available on-line (http://www.edesdeproject.eu/). See Table A1 (Appendix A) for DESDE’s code for the range of services in Australia.

### 2.4. Inclusion and Exclusion Criteria

Services had to meet the following criteria to be included in the present study:

(1) Providing specialised mental health services to people aged 65 years and over. Generic services for the general population, which were not specifically for, but could treat older people (e.g., general practitioners), were not included.
(2) Having temporal and organisational stability. Services with administrative support, dedicated space, finance and documentation to track activity, and stable funding were included. Those with less than three years funding were identified with an extension code “v” (variable). Care programmes that were clearly intended for a short period (less than three years) and for a specific reason were excluded.
(3) Being universally accessible, with no substantial out-of-pocket expenses.
(4) Providing care within the boundaries of the defined PHNs.

### 2.5. Data Collection

An online search, telephone directory search, and meetings with sector and peak bodies representatives were undertaken to identify and list all services providing mental health care to people aged 65 years and over in each study area. Next, data were collected from identified services through face-to-face interviews, telephone interviews or an online survey tool, between 2014 and 2017. Interviewers followed the DESDE-LTC service inventory questionnaire for the description and evaluation of mental health services, including location, administration, temporal stability, governance, and financing mechanisms. Data collection was conducted by researchers from the Glocal Integrated Mental Health Atlas project, composed of researchers from the Australian National University (the Mental Health Policy Unit), ConNectica (a social enterprise), University of Sydney and University of Loyola Andalucía (Spain) [32]. 

### 2.6. Data Analysis and Mapping 

The number of clinical teams (BSICs) and their Main Types of Care (MTCs) were analysed in every study area. We described: (a) availability of care: the service was operable upon demand for each 100,000 of the target population; (b) placement capacity: the maximum number of beds in residential care that was available at a given time per 100,000 of the target population; (c) balance of care: the percentage of health care services versus other care services; and (d) diversity of the care: the number of different categories of MTCs available in each study area. This study analysed all services that met the inclusion criteria in each study area (whole study population), but, in order to estimate the balance of care and the diversity of care for whole Australia, confidence intervals were also calculated (95% CI) considering the study areas as a representative sample.

The availability, placement capacity, balance of care and diversity of mental health services specifically for people aged 65 years and over were compared to the patterns of care for the other two main age groups: children and adolescents (population under 18 years old) and the general adult population (services available to all adults over the age of 18 years) following an heuristics approach to identify patterns of care and care gaps. The collection of these services followed the same method and the full information is available at the repository site [32]. Heuristics are simple decision aids that can be more accurate than other complex analytical tools [38]. They are transparent, speedy and not reliant on technology, and are particularly useful for conditions of uncertainty in decision making, including geriatrics [39].

### 2.7. Ethics Approvals

Data collection in all study areas was approved by the relevant ethics committees using the same standards of consent and confidentiality. For more details on the specific ethic approval for each study area please see the Australian National University Integrated Health Care Atlases repository site [32].

## 3. Results

### 3.1. Description of the Study Areas

The eleven study areas (PHNs catchment areas) included in this study cover 38.68% of the Australian continent and about 42% of the whole Australian population (Table 1). Most study areas have either a slightly lower or similar proportion of older people in their population than in Australia overall, except for Western New South Wales PHN, where the proportion is slightly higher. The proportions of older people in these areas altogether is less than the proportion in Australia as a whole (i.e., 14.18% vs. 15.41%) and include a total of 1,445,261 people, which is 38.13% of the Australian population aged 65 years and over. The average ageing index (the ratio of people aged 65 years and over to people younger than 15 years) in rural areas (79.81%) was higher than in urban areas (73.10%), and closer to the average in Australia (81.80%). 

The population density of the study areas shows the configuration of urban clusters and remote areas that represent Australia’s unique pattern of population density: a pattern characterised by both high urbanisation and high remoteness [40]. Regarding urban areas, the areas of lowest population density belonged to South Western Sydney, ACT and Perth South PHNs, with values below 200 people per square kilometres, and the highest belonged to Central and Eastern Sydney PHN, with a value of over 2500 people per square kilometres. The proportion of people with severe disability aged 65 years and over was lower than the national value in rural areas. In urban areas the proportion of people with severe disability was relatively different across the areas (from as low as 11.36% of people aged 65 years and over in Northern Sydney PHN to as high as 22.04% in South Western Sydney PHN) but the average (14.95%) was similar to the national value (14.34%). All study areas have similarly low unemployment rates. However, the Social-Economic Indexes for Area (SEIFA), which ranks areas in Australia according to relative socio-economic advantage and disadvantage, is slightly lower in rural areas than the average in Australia, while it is slightly higher in urban areas (except for South Western Sydney). 

### 3.2. Overview of Services in Urban Areas

#### 3.2.1. Availability of Care

A total of 35 care providers with 72 care teams (BSICs) were identified, across the urban areas, delivering 86 types of mental health care (MTCs) for older people, equivalent to 6.55 MTCs per 100,000 inhabitants aged 65 years and over. Health outpatient care was the most common type (48.8%). The majority of types of care (69.0%) were mobile outpatient services (outreach services), while 31.0% were hospital- or community centre-based outpatient care. This was followed by hospital (32.6%) and community (5.8%) residential care; information for care (5.8%); social outpatient care (3.5%), including 66.7% mobile and 33.3% non-mobile; day care (2.3%); and accessibility to care (1.2%). Self-help and voluntary services were not identified for this age group. 

**Figure 2:**
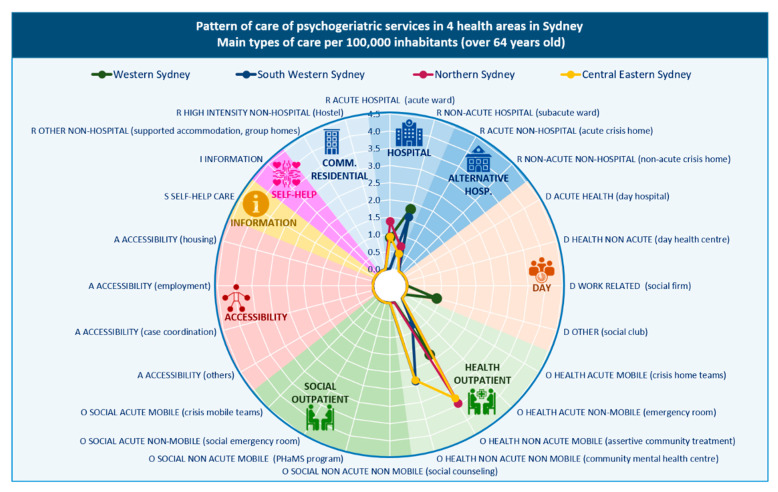
Pattern of care of mental health services in Sydney health districts: main types of care per 100,000 inhabitants aged 65 years and over.

**Figure 3:**
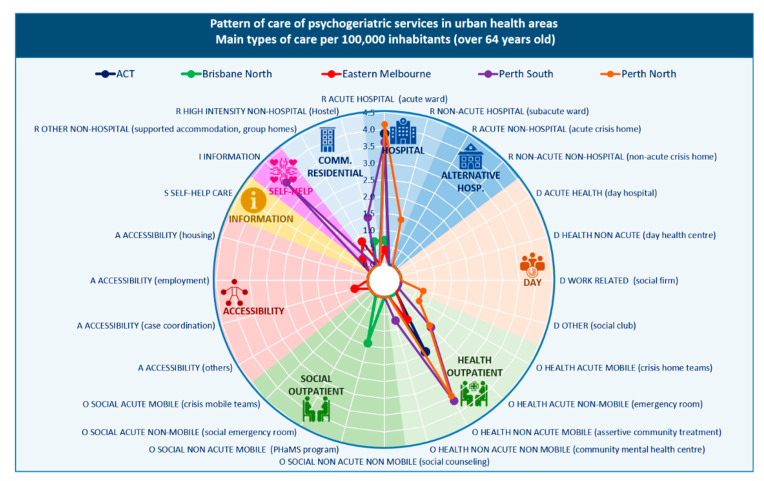
Pattern of care of mental health services in Australian Capital Territory, Brisbane, Melbourne, and Perth health districts: main types of care per 100,000 inhabitants aged 65 years and over.

Outpatient and residential services were identified in all urban areas. Five of the nine urban PHN catchment areas (ACT, Brisbane North, Central and Eastern Sydney, Northern Sydney, and South Western Sydney) only had these types of services. ‘Day care’ was available in Perth North and Western Sydney, ‘information for care’ was unique to Perth South, and ‘accessibility to care’ was unique to Eastern Melbourne. Care was limited in ACT to acute hospital (residential) and outreach outpatient care; and in Brisbane North to acute hospital and hostel care (residential) and social outreach support care (outpatient) (Figure 2 and Figure 3).

#### 3.2.2. Placement Capacity

The rate of specialised acute hospital beds was highest in the ACT, Northern Sydney and the Perth areas (over 20 beds per 100,000 residents over 65 years). Perth North and Northern Sydney also had the highest rate of beds in non-acute hospital care. In community residential care, Brisbane North, Eastern Melbourne and Perth South all had more than 10 beds per 100,000 residents over 65 years.

#### 3.2.3. Balance of Care

Brisbane North, Eastern Melbourne, Western Sydney and the two Perth areas provided both health- and non-health-related care. The remaining four urban areas provided exclusively health-related care.

#### 3.2.4. Diversity of Care

Nineteen different DESDE codes (representing the diversity of care) were identified in the urban PHNs explored (Table 2). This diversity of care was relatively lower than the diversity of care for the general adult population and for children, adolescents and young adults in these urban areas. 

### 3.3. Overview of Services in Rural Areas

#### 3.3.1. Availability of Care

A total of five care providers with 21 care teams were identified across the rural areas, delivering 24 types of mental health care (MTCs) for older people, equivalent to 18.01 MTCs per 100,000 inhabitants aged 65 years and over. Health outpatient care was the most common type (95.8%), the majority of which were outreach type care (82.6%), rather than hospital outpatient or community-centre outpatient based services (17.4%). Hospital residential care accounted for 4.2% of all available services. There were no services providing information for care, day care, accessibility to care, and self-help and voluntary care in rural areas. 

**Figure 4:**
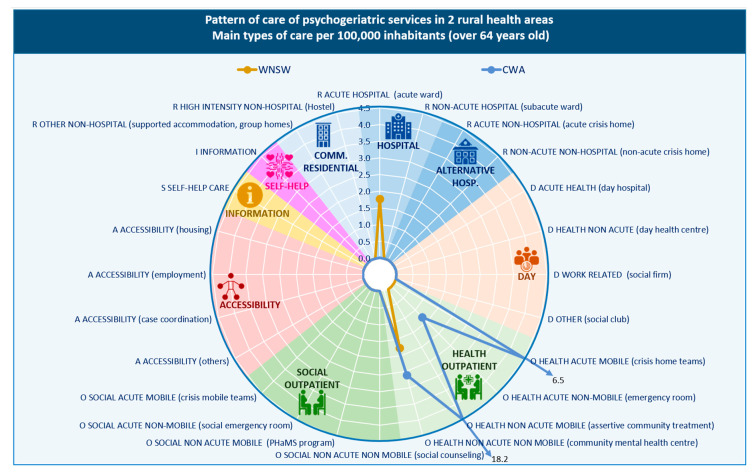
Pattern of care of mental health services in Western New South Wales and Central Western Australia rural areas: main types of care per 100,000 inhabitants aged 65 years and over. WNSW; western New South Wales, CWA; country Western Australia.

Outpatient care was the only type of care provided in CWA, while both outpatient and residential care were available in WNSW (Table 2). However, the outpatient care in CWA was more diverse, with acute (e.g., crisis home team) and community outreach (e.g., assertive community treatment) services the most commonly available types of care. In WNSW only acute hospital care and community mental health centre care (outpatient services) were available (Figure 4). 

#### 3.3.2. Placement Capacity

The bed rate of non-acute hospital care was six times higher than for acute care in WNSW (5.41 vs. 32.45 per 100,000 residents over 65 years).

#### 3.3.3. Balance of Care

Unlike our findings in urban areas, the only type of care available for older people in rural areas was health-related, with no social outpatient care identified.

#### 3.3.4. Diversity of Care

The diversity of care was limited to seven different types of care in these rural areas (Table 2). For the other age groups (adults and children, adolescents and young adults) the diversity of care in rural areas was higher. 

## 4. Discussion

This study aimed to describe the pattern of specialised mental healthcare provision (availability, capacity balance of care and diversity) for people aged 65 and over in selected health districts in Australia. Findings mainly indicate that: (1) service provision is mostly limited to hospital and outpatient care across all study areas; (2) outpatient services are generally limited to health-related outpatient care and there is a lack of social support care in all study areas; and (3) in rural areas hospital care is limited to acute hospital residential care, while in urban areas both acute and non-acute hospital care are mostly available. 

The pattern of service provision for older adults across study areas is generally similar to that previously identified for the general adult population in these areas [20,27]. However, the main difference is the lack of social support services, such as those which provide individual support to engage with the community or support to access community services, for this population across all study areas, with the exception of Brisbane North. This is important because social isolation and loneliness consistently emerge as risk factors of mental health in older people [41]. Older people are at particular risk of social isolation and perceived loneliness [42], so services providing social support from professionals such as social workers and case managers can make a key contribution to improving their mental health. The lack of these services across the areas studied, especially in rural areas, is an important issue which should be considered by policy makers and health system managers. 

Furthermore, consistent with patterns of care for general adult mental healthcare in urban and rural areas in Australia [20,21,27], there is a lack of diversity in service provision, most importantly in relation to day care services. Day care services provide a key link between inpatient/hospital care and outpatient and community care, particularly in urban areas. They provide a range of acute and non-acute types of care to people experiencing moderate mental health issues who do not meet criteria for inpatient admission, or who have just been discharged from the acute ward. Providing regular intervention before a crisis develops is one of their key features [43]. Additionally, the presence of day care services benefits family carers of older people with chronic mental conditions, receive significant benefit by reducing caregiver depression and caregiver burden [44,45]. The lack of some services such as information for care, which was lacking across all areas, may have lesser consequences because of the broad availability of online self-help tools and programs [46].

We found less diversity in the types of care available specifically for older adults than in those specifically for children, adolescents and young adults in the same areas. The proportion of services available for older people was also much smaller than that for younger populations, according to our study of mental health service provision in several Australian rural areas [20]. This last study also highlighted the underrepresentation of services for these two target populations considering their real weight in the overall population. This disproportion may be similar in urban areas, although new comparative studies are needed to obtain the complete picture. The disconnection between the supply of specific services and potential users, especially in the context of an ageing population, would deserves special attention from health administrations. 

While there was a general lack of diversity of service types across all study areas, the gap in services was greater in rural areas than in urban areas. In CWA, which covers 33% of the Australian continent, the only rural services available were those providing outpatient health care, both mobile and centre based, with mobile services the more frequently available of the two. Similarly, WNSW had a lower diversity of care with only two types of care acute hospital care and community mental health care available. It is also important to note that mental health services in rural areas for this population were much less available than those for the general adult population: adult hospital care, residential community care, social support care, limited day care, and accessibility to care services were all more available in general adult mental health care [20,27]. This suggests that mental health service provision to older people has not been a priority in rural areas, or that it has been difficult to build and staff mental health services for them. There is an ongoing debate on whether specialised services in rural and remote areas should be prioritised over more general care mainly relying on “augmented” primary care reinforced by specialists. The 2018 Senate enquiry on “Accessibility and quality of mental health services in rural and remote Australia,” recognised that rural access to quality mental health care is a pressing national issue, but no specific mention was made of mental health services for older people [47]. Similarly, the Orange Declaration on Rural and Remote Mental Health warned against “an over-emphasis on specialist and hospital services” in rural and remote care [48]. In any case, the standard description of patterns of specialised care in rural and remote areas, and its international comparison are urgently needed to gather local and national evidence that may inform and optimise models of care in rural areas.

This study has a number of limitations. First, we have not mapped the entire country, and the PHNs described here were not randomly selected. However, we have mapped 11 districts, or 42% of all PHNs in Australia. Although we have also mapped a 12th health district in Melbourne which had a similar pattern of mental health service provision to the other urban areas, it did not release its mental health Atlas and therefore was not included in the analysis. A major issue in decision analytics and organisational learning is when to stop gathering information to take a decision in real world conditions. In heuristics, “satisficing” refers to the point (threshold) at which obtaining more information becomes overly detrimental and costly [49]. The use of heuristics helps the decision maker to stop searching before this threshold has been crossed (optimal or minimum stopping time). The areas described in this analysis show a consistent pattern of care provision and include higher and lower resourced areas in urban and rural care in Australia. Given the proportion of areas analysed and the repetition of patterns identified, we estimate that the analysis of 11 areas provides a reliable scenario of older people’s mental health care in Australia.

In this case, the use of statistical analysis is limited due to the low number of units of the sample frame (31 Primary Health Districts in Australia). This is a typical problem in healthcare ecosystem research and it supports the use of heuristics and expert pattern recognition based on detailed and systematic description of the whole system of specialised care, rather than using significance of differences and power calculations across local areas. The problem of small numbers in organisational healthcare research has been revised by Sanders et al. [50] underscoring the limitations of samples below 100. Analytically, there are three broad strategies that can be taken when approaching the analysis of data with low sample sizes: a) maximise power using techniques specifically equipped for low sample; b) maximise the utility of available data and inference through permutation, resampling, or bootstrap methods; or c) use fuzzy logics for sensitivity analysis or undertake advanced modelling combining the other approaches. In any case the use of this information requires incorporating experts to improve, interpret and refine the results and provide better estimates [51]. This involves a multistep process where the standard description of the context, the patterns of care provision and utilisation, and the patterns of interventions and connections constitute the key components of the prior knowledge base for designing scenarios and running the model [18]. Furthermore, funding this type of research is even more complicated when we compare health systems instead of services or organisations. In Australia, the analysis of the Primary Health Network districts would require support and funding from the Department of Health (unfortunately not interested in this ecological approach to inform policy), and even then the units of analysis are still limited to 31. Under these restrictions we opted for a systems approach to heuristics to identify gaps and to elicit meaningful expert knowledge for consecutive modelling and informed decision making, as shown in previous studies [18]. The usability of this approach to heuristics under condition of high complexity and uncertainty has been reported previously by mental health planners in Spain and Australia [52,53]. Heuristics has also shown its usability in geriatric care [36].

Another limitation is that Atlases were collected over a time period of four years (2014–2017). Although services increase over time, previous longitudinal studies using the same method indicate that general patterns of care can be compared over this time span [54]. Finally, it is important to note that, ideally, the available information should be complemented with information on workforce characteristics, service utilisation and quality—which we have not attempted here. The present mapping is limited to services that are at least partially publicly funded, and are specific to people aged 65 and over. Therefore, fully private providers and generalized services such as general practitioners who treat older people were not included, and they should be mapped separately.

## 5. Conclusions

The pattern of care of mental health services available specifically for older people in the eleven Australian areas studied was characterised by a low diversity of care types, especially in rural areas; and a predominance of health-related care (i.e., hospital and outpatient care) over non-health (i.e., community residential and social support care). Moreover, we identified differences between rural and urban areas in hospital care with more diversified care available in the latter areas. Finally, the availability, capacity, balance of care and diversity of services for this age group is lower than that for children and adolescents, and for the general adult population. Thus, this study has detected care disparities and gaps between urban/rural areas and age groups that should be addressed by decision-makers. In order to understand whether these differences reflect inequities in care availability, relative technical efficiency analysis and comparative effectiveness analysis should be conducted using the exploratory data provided here [18].

The inclusion of study areas from different parts of Australia with different demographic, socioeconomic and health characteristics allowed us to identify the pattern of specialised mental health care for people aged 65 years and over across Australia. This will improve information on the local healthcare ecosystem and allow further comparison across Australia, and internationally. This can assist policy-makers in planning and resource allocation at local and national levels.

## Figures and Tables

**Table 1:** Key demographic and socioeconomic factors in study areas.

Indicators	ACT	Central and Eastern Sydney	Western Sydney	South Western Sydney	Northern Sydney	Brisbane North	Eastern Melbourne	Perth North	Perth South	Country WA	WNSW	Australia
Area (km^2^)	2351	626	766	6186	890	3901	3,956	2975	5148	2,477,561	433,379	7,594,238
Total population, 2016	411,667	1,599,658	975,083	989,536	928,456	1,003,843	1,531,395	1,062,621	981,218	531,613	307,905	24,592,907
Population aged 65 years and over 2015-2016 (%)	51,621 (12.54)	214,452 (13.41)	110,943 (11.38)	127,273 (12.86)	145,457 (15.67)	140,576 (14.00)	238,619 (15.58)	144,949 (13.64)	138,113 (14.08)	76,913 (14.47)	56,345 (18.30)	3,790,791 (15.41)
Density ratio, 2016	175.10	2555.36	1272.95	159.96	1043.21	257.33	387.11	357.18	190.60	0.21	0.71	3.19
Ageing index, 2016 (%)	66.06	88.43	54.07	60.22	84.71	74.96	85.33	71.96	72.19	70.25	89.37	81.80
Unemployment, 2016 (%)	4.48	4.22	5.98	6.32	3.97	5.19	4.75	5.80	6.26	5.76	5.70	5.88
Low income households (households in bottom 40% of income distribution), 2016 (%)	40.41	31.90	38.12	46.62	24.52	34.65	36.63	39.06	44.34	47.66	49.26	40.54
SEIFA Index of Relative Socio-economic Disadvantage (based on Australian score of 1000), 2016	1075	1036	1005	945	1093	1032	1048	1039	1013	976	954	1000
People 65 years and over with a profound or severe disability and living in the community, 2016	13.67%	16.15%	17.98%	22.04%	11.36%	13.48%	13.74%	12.55%	13.34%	11.56%	12.65%	14.34%
Residential aged care places per 1,000 population aged 65 years and over, 2016	47.91	58.03	45.83	52.71	61.36	50.56	55.55	45.11	49.37	41.13	61.02	52.61

Sources: Social Health Atlases of Australia, 2016; and Australian Bureau of Statistics, 2016; SEIFA: Socio-Economic Indexes for Areas; ACT: Australian Capital Territory; WA: Western Australia; WNSW: Western New South Wales.

**Table 2:** Diversity, pattern and balance of older people’s mental health main types of care in the study areas.

Study Areas	Urban Study Areas										Rural Study Areas			Australia
	Brisbane North	ACT	Eastern Melbourne	Central & Eastern Sydney	Northern Sydney	South Western Sydney	Western Sydney	Perth South	Perth North	Total	Country Western Australia	Western NSW	Total	Total Number/Estimation (95% CI)
Mental health service provision (raw numbers)														
Number of MTC	4	3	7	15	8	6	6	20	17	86	22	2	24	110
Diversity of MTC in older people (>64)	3	2	6	5	3	3	5	6	7	19	6	2	7	19
Diversity of MTC in the general adult population (>18)	31	29	39	48	32	26	29	32	35	69	25	24	36	71
Diversity of MTC in children, adolescent and young adults (<25)	6 ^†^	12	20	7 ^‡^	8	4	7	15	8	33 ^†,‡^	11	6	13	34
Balance of care (percentage of total MTC)														
Health care (%)	25.0	100.0	42.9	100.0	100.0	100.0	83.3	65.0	94.1	81.4	100.0	100.0	100.0	85.5 (78.9–92.0)
Other care (%)	75.0	0.0	57.1	0.0	0.0	0.0	16.7	35.0	5.9	18.6	0.0	0.0	0.0	14.5 (8.0–21.1)
Care classification (percentage of total MTC)														
Hospital (%)	25.0	66.7	14.3	20,0	37.5	33.3	50.0	25.0	47.1	32.6	0.0	50.0	4.2	26.4 (18.1–34.6)
Community residential (%)	25.0	0.0	28.6	0.0	0.0	0.0	0.0	10.0	0.0	5.8	0.0	0.0	0.0	4.5 (0.7–8.4)
Day care (%)	0.0	0.0	0.0	0.0	0.0	0.0	16.7	0.0	5.9	2.3	0.0	0.0	0.0	1.8 (−0.7–4.3)
Health outpatient (%)	0.0	33.3	28.6	80.0	62.5	66.7	33.3	40.0	47.1	48.8	100.0	33.3	95.8	59.1 (49.9–68.3)
Social outpatient (%)	50.0	0.0	14.3	0.0	0.0	0.0	0.0	0.0	0.0	3.5	0.0	0.0	0.0	2.7 (−0.3–5.8)
Info. (%)	0.0	0.0	0.0	0.0	0.0	0.0	0.0	25.0	0.0	5.8	0.0	0.0	0.0	4.5 (0.7–8.4)
Access. (%)	0.0	0.0	14.3	0.0	0.0	0.0	0.0	0.0	0.0	1.2	0.0	0.0	0.0	0.9 (−0.9–2.7)

ACT; Australian Capital Territory, MTC; Main Type of Care, n-mob; Non-mobile outpatient care, mob; Mobile outpatient care, Info; information for care, Access; Accessibility to care. ^†^ Only services for transition to adulthood in Brisbane North; ^‡^ Only services in South Eastern Sydney LHD in Central Eastern Sydney.

## Appendix A

**Table A1:** Glossary of DESDE-LTC Main types of care (MTC), descriptions and common terms.

Broad Category	Description	Other Common Terms	Main Type of Care (MTC)
**Residential**	Facilities which provide beds overnight for users for a purpose related to the clinical and social management of their health condition	Accommodation, Hospital, Residential	R
Hospital	ACUTE. Users are admitted to hospital typically within 24 h because of their crisis condition. Surveillance level and length of stay varies depending on the code	High Dependency Inpatient; Acute Care Unit; Intensive Care Unit; Psychiatric Assessment and Planning Unit; Specialised Acute Mental Health Units for Older People or Specialised Hospitals for Older People	R1–R3
Hospital	NON-ACUTE. Facilities which do not satisfy acute conditions. It can be time limited or indefinite depending on the code.	Sub-acute; Community Care Units; Extended Care Mental Health Rehabilitation Unit; Extended Treatment	R4, R6
Alternative to hospital	ACUTE. Facilities providing acute residential care outside the location of a registered hospital	Crisis homes	R0, R3.1
Alternative to hospital	NON-ACUTE. Facilities with 24 h medical support on site. It can be time limited or indefinite depending on the code	Therapeutic Communities	R5, R7
Community	HIGH INTENSITY. Facilities with 24 h (non-medical) support. Length of stay (4weeks to indefinite) varies depending on the code.	Step up-Step Down (SUSD); Prevention and Recovery Care (PARC); Rehabilitation residences; Supported accommodation; Specialised Residential Aged Care	R8, R11
Community	MEDIUM AND LOW INTENSITY. Facilities with a range of support that varies from daily to fewer to 5 days a week depending on the code. Length of stay (4 weeks to indefinite) varies depending on the code.	Psychiatric Hostel; Group Houses; Supported Accommodation	R9, R10, R12, R13
**Day services**	Facilities available to several users at a time that provide some combination of planned treatment for users’ needs, with regular opening hours during which they are normally available, and expect users to stay at the facilities beyond the periods during which they have face-to-face contact with staff.	Day services	D
Day	ACUTE HEALTH. Users are admitted to the service to because of their crisis condition. Admittance varies typically from 72 h to 4 weeks, depending on the code	Day Hospital services (non-existent in Australia)	D0–D1
Day	NON-ACUTE HEALTH. Typically, at least 20% of staff are qualified health professionals with at least a four year university degree. Depending on the code it can be high (equivalent to 4 half days) or low intensity	Recovery Services; Rehabilitation Services, Therapeutic Day services, Outpatient ECT services,	D4.1, D8.1
Day	WORK-RELATED. Facilities which provide users with the opportunity to work. The salary varies depending on the code: normal wage; 50% of typical wage; not paid or symbolic pay.	Disability Enterprises; Social firms; Workers Coop; Occupational centres; Integration workplace; sheltered work	D2–D3, D6–D7
Day	OTHER. Facilities providing education, social or other non-health-related care. Depending on the code it can be high (equivalent to 4 half days) or low intensity. Structured (activities available more than 25% o opening hours) or non-structured.	Social Clubs; Club Houses; Vocational training; psychiatric drop-in centre, Day centres	D4.2–D4.4, D8.2–D8.4, D5, D9, D10
**Outpatient**	Facilities providing contact between staff and users for some purpose related to management of their condition that are not provided as a part of delivery of residential or day and structured activity care teams, as defined below.	Community or ambulatory care; psychosocial support	O
Health	ACUTE MOBILE. The service provides assessment and initial treatment in response to a health-related crisis, typically same day response during working hours or at least within 72 h after the care demand. At least 50% of contacts take place outside the service (e.g., user’s home). Depending on the code it can be 24 h or limited hours.	Crisis and Assessment Teams; Assertive Community Treatment	O1.1, O2.1
Health	ACUTE NON-MOBILE. The service provides assessment and initial treatment in response to a health-related crisis, the purpose is to treat the user in the service, in no case mobile attention exceeds 50% of overall activity. Depending on the code it can be 24 h or limited hours.	Emergency Units or Depts, Psychiatric Emergency; Psychiatric Liaison	O3.1, O4.1
Health	NON-ACUTE MOBILE. The service does not fulfil criteria for acute care. At least 50% of contacts take place outside the service (e.g., user’s home). Depending on the code it can be high intensity (3 times/week), medium intensity (once a fortnight), low intensity (once a month or less)	Mobile Support and Treatment Team; Community Outreach, Specialised Community Mental Health Teams for Older People	O5.1, O6.1, O7.1
Health	NON-ACUTE NON-MOBILE. The service does not fulfil criteria for acute care. The purpose is to treat the user in the service, in no case mobile attention exceeds 50% of overall activity. Depending on the code it can be high intensity (3 times/week), medium intensity (once a fortnight), low intensity (once a month or less)	Outpatients; Specialised Outpatient Clinics for Older People; Clinic services, Dual Diagnosis; Community Care/Continuing Care,	O8.1, O9.1, O10.1
Social	NON-ACUTE NON-MOBILE. As in non-acute non mobile health but providing other type of care different than health (social, work)	Daily Living, Living Skills Development or Support eg: Art therapy classes, financial or budgeting support (centre based)	O8.2, O9.2, O10.2
Social	NON-ACUTE MOBILE. As in non-acute mobile health but providing other type of care different than health (social, work)	Personal Helpers and Mentors; Psychosocial outreach support	O5.2, O6.2, O7.2
Social	ACUTE NON-MOBILE. As in acute non mobile health but providing other type of care different than health (social, work)	Family and sexual violence crisis services	O3.2, O4.2
Social	ACUTE MOBILE NON-HEALTH. As in acute mobile health but providing other type of care different than health (social, work)		O1.2, O2.2
**Accessibility**	Facilities which main aim is to facilitate accessibility to care for users with a specific condition		**A**
	Services that facilitates the access to information; Services that facilitates physical mobility; services that facilitates personal accompaniment; Services that facilitates case coordination; Services that facilitates access to employment or housing.	Partners In Recovery (now ceased), Tenancy Support	A1–A5
**Information**	Facilities that provide users from the defined target group with information and/or an assessment of their needs. Does not entail subsequent monitoring/follow-up or direct care provision		**I**
	Guidance and assessment. Information	Telephone triage; Intake & Assessment; Support helplines; Lifeline; Hotline, Information services; Leaflets; Websites	I1–I2
